# Patient experiences with tissue‐based genomic testing during active surveillance for prostate cancer

**DOI:** 10.1002/bco2.277

**Published:** 2023-08-15

**Authors:** Michael S. Leapman, Ryan Sutherland, Cary P. Gross, Xiaomei Ma, Tyler M. Seibert, Matthew R. Cooperberg, William J. Catalona, Stacy Loeb, Dena Schulman‐Green

**Affiliations:** ^1^ Department of Urology Yale School of Medicine New Haven Connecticut USA; ^2^ Yale Cancer Outcomes Public Policy, and Effectiveness Research Center New Haven Connecticut USA; ^3^ Department of Chronic Disease Epidemiology Yale School of Public Health New Haven Connecticut USA; ^4^ Yale School of Medicine New Haven Connecticut USA; ^5^ Department of Internal Medicine Yale School of Medicine New Haven Connecticut USA; ^6^ Department of Radiation Medicine and Applied Sciences University of California San Diego La Jolla California USA; ^7^ Department of Radiology University of California San Diego La Jolla California USA; ^8^ Department of Bioengineering University of California San Diego La Jolla California USA; ^9^ Department of Urology University of California San Francisco San Francisco California USA; ^10^ Department of Epidemiology and Biostatistics University of California San Francisco San Francisco California USA; ^11^ Department of Urology Northwestern University Feinberg School of Medicine Chicago Illinois USA; ^12^ Departments of Urology and Population Health New York University Langone Health New York New York USA; ^13^ Manhattan Veterans Affairs Medical Center New York New York USA; ^14^ New York University Rory Meyers College of Nursing New York New York USA

**Keywords:** active surveillance, biomarkers, decision‐making, genomic testing, prostate cancer, qualitative

## Abstract

**Background:**

Tissue‐based gene expression (genomic) tests provide estimates of prostate cancer aggressiveness and are increasingly used for patients considering or engaged in active surveillance. However, little is known about patient experiences with genomic testing and its role in their decision‐making.

**Methods:**

We performed a qualitative study consisting of in‐depth, semi‐structured interviews of patients with low‐ or favourable‐intermediate‐risk prostate cancer managed with active surveillance. We purposively sampled to include patients who received biopsy‐based genomic testing as part of clinical care. The interview guide focused on experiences with genomic testing during patients' decision‐making for prostate cancer management and understanding of genomic test results. We continued interviews until thematic saturation was reached, iteratively created a code key and used conventional content analysis to analyse data.

**Results:**

Participants' (*n* = 20) mean age was 68 years (range 51–79). At initial biopsy, 17 (85%) had a Gleason grade group 1, and 3 (15%) had a grade group 2 prostate cancer. The decision to undergo genomic testing was driven by both participants and physicians' recommendations; however, some participants were unaware that testing had occurred. Overall, participants understood the role of genomic testing in estimating their prostate cancer risk, and the test results increased their confidence in the decision for active surveillance. Participants had some misconceptions about the difference between tissue‐based gene expression tests and germline genetic tests and commonly believed that tissue‐based tests measured hereditary cancer risk. While some participants expressed satisfaction with their physicians' explanations, others felt that communication was limited and lacked sufficient detail.

**Conclusion:**

Patients interact with and are influenced by the results of biopsy‐based genomic testing during active surveillance for prostate cancer, despite gaps in understanding about test results. Our findings indicate areas for improvement in patient counselling in order to increase patient knowledge and comfort with genomic testing.

## 1 INTRODUCTION

Uncertainty about cancer aggressiveness is a prominent barrier to the selection and adherence to active surveillance for low‐grade prostate cancer.^1^ Several tissue‐based (‘genomic’) tests measuring mRNA expression of genes associated with prostate cancer aggressiveness provide prognostic estimates such as the probability of metastasis or death.^2^ Genomic testing is available at multiple junctures in the disease continuum, particularly for patients with low‐ and intermediate‐risk prostate cancer who are considering or enrolled in active surveillance.^3^, ^4^ In this subset, there is accumulating evidence that genomic testing can identify patients at higher or lower risk for disease reclassification, informing the appropriateness or intensity of monitoring.^5^ However, it is unclear whether genomic testing exerts intended impacts on patient decision‐making. For example, in a randomized trial, use of a 12‐gene assay was associated with less use of active surveillance, particularly among patients with lower health literacy.^6^ Thus, it remains unknown if genomic testing is empirically effective at improving the utilization, adherence and clinical outcomes of active surveillance.^7^

Despite growing use, little is known about patients' perspectives on or experiences with genomic testing.^8^ As active surveillance has become the dominant strategy for patients with low‐risk prostate cancer, a deeper understanding of how patients perceive and interact with genomic testing is needed for several reasons.^9^ It is unknown whether testing alleviates cancer‐related uncertainty and could be used to reduce over‐treatment of low‐risk prostate cancer. Appreciating how genomic testing is incorporated into patient decision‐making is crucial to understanding its effectiveness as an intervention. Moreover, these expensive, patient‐facing products are laden with medical and statistical jargon (e.g. risks of metastasis, mortality and 95% confidence intervals), introducing the possibility that testing may sow confusion or increase cancer‐related anxiety, which are antecedents of over‐treatment.^10^, ^11^, ^12^ Given the relative novelty of these tests, there is little independent guidance for how providers should communicate genomic testing results to meet patients' informational needs.

The purpose of this study was to explore the perceptions and experiences of patients with prostate cancer managed with active surveillance who underwent tissue‐based genomic testing. We sought to elicit patients' overall awareness and perceptions of genomic testing, understand the impact of testing on decision‐making and identify barriers to effective communication with potential strategies for practice improvement. Greater awareness of patient informational needs may enhance the effectiveness of testing by improving decisional quality and may reduce anxiety and uncertainty.

## 2 METHODS

### 2.1 Sample selection and patient enrolment

We conducted a qualitative descriptive study of patients with prostate cancer who had elected active surveillance as initial management. We conducted this analysis within a larger qualitative descriptive study that examined the experiences of patients managed with active surveillance who underwent assessment with new diagnostic technologies, including both prostate magnetic resonance imaging (MRI) and genomic testing. Patients were enrolled from a regional healthcare delivery network of academic and community‐affiliated clinics and hospitals in the Northeastern United States. The study investigators screened patient medical records for clinical eligibility (low‐ and intermediate‐risk prostate cancer, receipt of prostate MRI and genomic testing). Patients were then contacted by a study coordinator to further screen for eligibility, including confirmation of continued active surveillance monitoring, English‐language fluency and interest. Purposive sampling was conducted to ensure representation of racial and ethnic minorities historically under‐studied in prostate cancer, including Black and Hispanic or Latino‐identifying individuals,^13^ and to ensure representation of patients from academic, community‐affiliated practices across the age and health insurance spectrum.

Participants were informed that their participation was voluntary and were offered a $50 gift certificate as compensation for their time. All interviews were conducted via Zoom or telephone call based on patient preference. Video interviews were recorded and exported using Zoom, and telephone interviews were audio recorded. The audio recordings were then transcribed into text, which served as the primary data for analysis. This study was approved by the Yale University Institutional Review Board.

### 2.2 Interview guide

Study team members (ML, RS, DSG) developed an interview guide (Table 1) aimed at eliciting patients' perspectives and experiences about new risk assessment technologies for localized prostate cancer using open‐ended questions with follow‐up probes as needed. The interviews were designed to explore the participants' perceptions and experiences of tissue‐based genomic testing, including their decision‐making process, understanding of test results, the impact of test results on their treatment decisions, communication experiences and preferences, overall satisfaction with the testing experience and potential strategies for practice improvement.

**TABLE 1:** Interview guide for descriptive study of patients undergoing genomic testing as a component of active surveillance for prostate cancer.

1	We are interested in learning about how new types of scans and tests are used to help patients diagnosed with prostate cancer make decisions about whether they should have their cancer watched (‘active surveillance’) or treated. Prostate MRI and genomic testing are two new forms of testing that are designed to give more information about how aggressive a man's prostate cancer is. As a result, they can help patients make an informed decision about being treated treatment or having active surveillance. Thinking back on your experience, what was your approach and decision‐making process?
2	Deciding about active surveillance or treatment for prostate cancer usually involves discussion about the risks and benefits of each approach. Can you tell me about how information about risk was presented to you? (Probe: Did you find it to be effective? Was there anything that you found to be confusing?)
3	I understand that your doctors ordered a genomic test from your prostate biopsy. What is your understanding of why genomic testing was done and what it showed? (Probe: How did your doctor explain the results? Do you have any unresolved questions about your test results?)
4	We are interested in learning how communication about genomic testing could be improved during decision‐making for patients. How do you think that the information about genomic testing could have been better explained to you?
5	How do you think that the information about your genomic testing could be better communicated by your doctor? (Probe: Does hearing a percentage [for example an X% risk of cancer metastasizing] help you? Or would you prefer to see this information visually? Would it be more helpful for your doctor to describe risks using words like ‘high’ or ‘low’?)
6	How would you feel about using an electronic survey to help your doctor understand your preferences about discussing risk estimates? Would you want to complete this before your visit with your doctors or discuss it with them directly?
7	As you know, active surveillance involves close monitoring of prostate cancer. Is having a prostate MRI/genomic testing a component of your monitoring plan? If so, do you think the role of these tests in the monitoring process has been explained in an effective way to you? If not, how could it be improved? Do you think a written or electronic document to track this information over time would be helpful?
8	Is there anything we have not discussed that you think it is important for us to know about how new technologies can help patients with low‐risk prostate cancer with decision‐making?

### 2.3 Data analysis

Interviews were transcribed verbatim, reviewed for accuracy and uploaded to the Dedoose software platform. The study team performed conventional content analysis, an inductive method used when the intent is to describe a phenomenon about which there is limited literature.^14^, ^15^ Interviewing and data analysis were concurrent. First, the study coders (RS and ML) independently reviewed each study transcript to gain familiarity. Second, the coders performed independent line‐by‐line coding on each transcript to identify initial coding categories. Next, in joint session, the codes' meanings were specified, codes were named using language derived from the transcripts, and codes were classified within larger thematic categories. Last, the coders identified exemplars for each code. This process occurred iteratively until all transcripts were coded, and the final code key was applied to all transcripts.^15^, ^16^

## 3 FINDINGS

Fifty‐eight patients were screened and approached for participation, and 20 met criteria and agreed to participate until thematic saturation was reached. Participants' mean age was 68 years, 17 (85%) had a Gleason grade group 1, and three (15%) had a grade group 2 disease at diagnosis. Fourteen (70%) participants identified their race/ethnicity as White, five (25%) as Black and two (10%) as Latino. The mean interview length was 48 min. Seventeen interviews (85%) were conducted via Zoom and three (15%) by telephone. Themes were identified relating to the perception (drivers of the decision to undergo genomic testing, understanding of genomic testing results, impact of testing on confidence in active surveillance) and experiences (communication of genomic testing results, patient‐facing materials) of genomic testing. We expand on each below.

### 3.1 Patient perceptions of genomic testing

#### 3.1.1 Drivers of the decision to undergo genomic testing

Participants were usually made aware of genomic testing by their physicians. In some instances, the decision to undergo genomic testing was offered as an option:

[My doctor] basically put it out there as an alternative for me to consider as a way of determining getting a closer view as to how aggressive the actual cancer was… by looking at the cells, I believed that we would have a better fix on are they really aggressive cancer cells or not. (Participant 4)

For others, the decision was made unilaterally by physicians: ‘… He [my doctor] ordered it because the results that came back were a little bit more advanced than my first time around’ (Participant 19).

In some instances, participants were unaware that testing had occurred: ‘What is the genomic testing? I was [tested]—No? I've never heard anything of that … never even mentioned to me, no’ ( Participant 14).

#### 3.1.2 Understanding of genomic testing results

While participants understood the role of genomic testing as a tool to estimate prostate cancer risk, some did not appreciate the difference between tissue‐based gene expression tests and germline genetic tests and commonly believed that tissue‐based tests measured hereditary cancer risk.

The misperception that tissue‐based gene expression testing measures inherited features may lead to the belief that disease progression is unlikely or impossible. Some participants held beliefs about genetics as deterministic of health and prostate cancer outcome:

So I have been under the impression that it's a one‐time test, I'm good to go, I have good genes and this cancer is not going to affect me. So therefore, I've ignored the genomic testing and assumed I never have to have it done again. (Participant 8)

Some participants misunderstood that the motivation for repeat genomic testing was to obtain additional tissue for analysis. Potential concerns for spatial transcriptomic heterogeneity and sampling effects were not raised.

I don't know if the second genomic test can present different results. I assume that because the test has been around for a year and a half later from the first genomic test, that they have more data to work with. But the DNA shouldn't change, you would think. My assumption is the DNA is the DNA. (Participant 3)

Another participant added:

I'm also a believer that genetics are much more important than all the berries and palm juice you can drink. Genetics are showing you the indicators when they look at it, and they see these different little things that you are dedicated to having the possibility that it may go this way as opposed, if the numbers were the other way around. (Participant 1)

#### 3.1.3 Impact of testing on confidence in active surveillance

Test results increased participants' confidence in the decision of active surveillance. Patients generally found low, discrete predictions of mortality risk to be reassuring of the decision to undergo active surveillance:

The genomic test, more than any other test, that's the one that I trusted, and that's how I came to the conclusion [for active surveillance]. And I could tell, Dr. [X] gave me the feeling that that was his recommendation for active surveillance. But I needed something in addition, and that genomic test, one and a half percent of risk of mortality. (Participant 3)

Some participants found that favourable predictions were encouraging even if they were uncertain about the derivation of estimates:

I mean, it's just 2% over 10 years. To die from it means it's a pretty low probability, i.e., low risk. So that simple one sentence was very helpful in putting it all into context … The risk assessment is very clear, right? 2% of dying in 10 years, I thought that was very clear. So the assessment itself or the results were clear, but the mechanics behind it are less clear. (Participant 8)

Agreement across multiple clinical parameters was reassuring to participants that active surveillance was an appropriate management strategy:

It's a data point that has some degree of predictability. My understanding of this particular genomic testing is it's not exactly intended for what we're using it for. It's to some extent being used for dosage for other kinds of treatment, but it still has a good risk assessment component, and that's what we used it for. Again, it's just one more data point. If you're going to have a plan, you should have some data points. (Participant 5)

Based on impressions from their physicians, some participants expressed favourable views of genomics as a tool that can be used to dictate the intensity of invasive active surveillance monitoring procedures, particularly prostate biopsy:

I really didn't love the idea of getting a biopsy every year. So with the idea that that could make it less frequent I was like, “Great. Do the test. Let's see where this takes us.” And the first one did bring it back to where I didn't need a biopsy every year. I was down to every two years, which was great news to me. (Participant 6)

Other patients held more circumspect views of the prognostic value of genomic testing, appreciating that their results were not deterministic and carried uncertainty:

I've researched genomic testing … and it's pretty rambly information. You can read different things [but it] isn't [an] open and shut case on how valid it is. And that's because it's so new. So at this point, no one's able to say, ‘Look, man, I guarantee you're not going to get a more severe level of cancer because this genomic testing is so over the validity of it’. They don't have those answers yet. (Participant 9)

### 3.2 Experience of genomic testing

#### 3.2.1 Communication of genomic testing results

Participants conveyed a spectrum of informational and communication needs regarding genomic testing results. Patients with greater numeracy or quantitative backgrounds expressed a preference for more detail when discussing results, such as probabilities and visual representations of data: ‘And for me, as an engineer, it was straightforward. I understood it, I saw the curves, I saw the data, I understood the probabilities. So for me, it was a no‐brainer, it was fine, it was great’ (Participant 8).

While some participants expressed satisfaction with the explanations provided by their physicians, others felt that communication was limited or lacked sufficient detail. Some patients expressed reluctance to ask their physicians for more information about genomic testing:

As far as the genomic testing, I never really got much information about that at all. Only, ‘Hey, your score is, low probability of like three out of 10 or something’. And that's the long and short of it. I do my own research, which is why I don't bug the doctor. (Participant 9)

Others highlighted the importance of providers recognizing the potential impact of genomic testing results: ‘I think it's important [to] reveal results in person in an empathetic way, and not just send a report that has implications’ (Participant 3).

#### 3.2.2 Patient‐facing materials

Many patients did not directly view or read genomic testing results. However, patients generally viewed reports as difficult to read, and presenting details in small font. Densely written materials may obscure clinical messaging to patients. One participant summarized, ‘It's very hard to read. I mean just cosmetically, it's just it's all mushy and terrible’ (Participant 5). Another added, ‘But I just recall reading some fine print on the back page. I just remember it being two pages from Decipher and reading the fine print’ (Participant 3).

### 3.3 Clinical practice implications

Table 2 presents a summary of themes and concerns raised by patients as well as data‐indicated strategies to address them. For example, for the theme of drivers of the decision to undergo genomic testing, the patients identified the barrier of being unaware that testing was performed, or not being asked about genomic testing of biopsy specimens. The study team's recommended strategies to address this barrier are to communicate that genomic testing is optional, to set expectations about how testing could impact clinical management and to confirm patient understanding that testing will be conducted.

**TABLE 2:** Summary of themes and communication barriers expressed by patients with potential strategies for clinical improvement.

Theme	Barrier identified	Interview‐informed strategy for practice improvement
Drivers of the decision to undergo genomic testing	Patients unaware or not asked about genomic testing of biopsy specimens	Communicate that genomic testing is optional Set expectations about how testing could impact clinical management (e.g. influencing decision for active surveillance, intensity of surveillance monitoring) Confirm patient understanding that testing will be conducted
Understanding of genomic testing results	Misunderstanding of genomic testing results as measuring hereditary cancer risk	Emphasize that tissue‐based gene expression tests do not measure hereditary risk and that profiles may change over time Communicate that disease progression is possible despite favourable genomic test result; unfavourable results do not guarantee adverse outcome with active surveillance
Impact of testing on confidence in active surveillance	Uncertainty about how genomic profiles relate to appropriateness of active surveillance	Communicate cancer mortality estimates Present genomic testing results in the context of other clinical parameters Discuss limitations of genomic testing in active surveillance (e.g. absence of evidence supporting genomic tests as prognostic for long‐term disease reclassification or clinical outcome of surveillance)
Communication of genomic testing results	Informational needs and understanding vary among patients	Assess patient numeracy and preferences about level of detail in discussion (e.g. ‘how much of the specific details about this test would you like to know?’) Prioritize straightforward qualitative descriptions (high vs. low) over numerical estimates with low numeracy Provide opportunities for patient questions (e.g. ‘Do you have any questions about your genomic testing results?’)
Patient‐facing materials	Genomic testing reports in small font, use statistical and medical jargon that are unfamiliar to patients	Commercial genomic testing reports may be improved by incorporating patient‐directed reports that include larger text and plain language summaries

## 4 DISCUSSION

In this qualitative study of patients with prostate cancer managed with active surveillance, we uncovered a range of perceptions and experiences regarding tissue‐based gene expression testing. In general, patients held favourable views of the information and overall experience of genomic testing and understood its role as an estimate of cancer aggressiveness. We also identified common misperceptions and information gaps. In particular, patients often conflated genomic testing with germline genetic testing and perceived favourable mRNA expression results from a single biopsy as deterministic of their long‐term outcome. Notably, some patients were unaware that genomic testing had been conducted, precluding value as a patient decision tool and raising questions of resource overuse.

There are several notable findings from this work that can inform a framework for understanding of the role of genomic testing during active surveillance of low‐grade prostate cancer. Overall, patients held favourable views of genomic testing and recognized their position as risk stratification tools, in some instances characterizing these tests as central to their selection of active surveillance. Prior qualitative studies have found that a patient's personal assessment of risk is a major determinant in active surveillance decision making.^17^ Our findings imply that genomic testing can directly contribute to perceptions of risk in patients who are aware of the results and implications.^11^ Therefore, genomic tests may constructively address uncertainty as a barrier to active surveillance selection for some patients, particularly when results suggest low absolute risks.^18^ Conversely, genomic testing may have little value or present risks for patients who do not receive an explanation or do not adequately understand their results by conveying unrealistic expectations that surveillance monitoring is not necessary. Importantly, some participants were unaware that testing had occurred, suggesting absent or ineffective communication. These findings could also reflect routine testing by some providers or limitations of patient recall but nonetheless underscore the importance of direct discussions about both the decision to pursue genomic testing and test results for them to contribute to shared decision‐making.

The nature of communication between patients and their physician was central to the selection and interpretation of genomic testing. In our sample, the option to pursue genomic testing was often raised by or entirely driven by providers. These findings are consistent with other work that has identified patient–provider relationships and trust as a key component and facilitator of selecting active surveillance.^19^, ^20^ Although genomic testing reports are patient‐facing, we found that patients often look to their physicians for interpretation, particularly with regard to their practical clinical meaning. When reflecting on these discussions, patients expressed a preference for direct and concise information about genomic testing. Providers should take care to communicate simple and clear messages to most patients, for example, a ‘very low risk of death from prostate cancer’, rather than complex predictions or surrogate endpoints such as probabilities of pathologic upgrading or upstaging.

Improving information flow is a priority for patients enrolled in active surveillance, but virtually no guidance has been available to inform how best to communicate genomic testing results with patients.^21^, ^22^ We uncovered a range of information needs when deciding on and interpreting the results of genomic testing. Overall, patients expressed a preference for learning more about genomic testing results as there were frequently questions or details that remained unanswered. Due to their technical complexity, patients approach genomic testing results with widely different understanding and preferences for information. For example, we found, as have others, that some subjects with familiarity with quantitative information gravitated towards more detailed elements of reporting.^23^ While for others, more simplistic explanations are sufficient and may be optimally effective at communicating pertinent clinical information. These findings indicate that effective communication about genomic test results should be highly personalized but prioritize clarity and simplicity, especially if patient‐facing materials are delivered electronically.^24^, ^25^

A notable finding from this analysis is that the results from gene expression assays are often believed to reflect germline genetics. As a result, patients may hold the misperception that their prostate cancer risk is static and that disease progression or reclassification is not possible, generating confusion or undermining future monitoring. This finding is in line with prior studies which found that patients generally hold deterministic views of genetics in overall health and prostate cancer.^26^ To respond to this information gap, providers should employ clear language to convey that commercial genomic tests measure gene expression levels, which, themselves, may change over time or with resampling. Importantly, approximately one‐quarter of surveyed urologists also incorrectly reported that gene expressed tests measured hereditary cancer risk, highlighting the need for these efforts to be combined with provider education.^27^

### 4.1 Study limitations

There are limitations to this study. These findings reflect the context and experiences of study participants and are not broadly representative of all patients with favourable‐risk prostate cancer. We took explicit measures to include patients from diverse clinical and demographic contexts—specifically based on race/ethnicity, cancer risk strata and academic versus community or VA affiliation. However, these findings are limited to our sampling frame that was skewed towards patients managed in an academic tertiary referral centre. Because our sample included patients who selected active surveillance, additional study is needed to evaluate the experiences of patients in whom genomic testing may have contributed to immediate treatment or early discontinuation of active surveillance.

In addition, as a result of the Covid‐19 pandemic, all study interviews were conducted remotely. Although the use of teleconferencing software for qualitative research has been increasingly studied, this is relatively new approach, raising the possibility that we missed non‐verbal cues or insufficiently developed rapport compared with in‐person interviews.^28^ Nevertheless, this study is the first to examine patient experiences with genomic testing during active surveillance. Our participants' reports can be used to enhance the quality of counselling and decision‐making among patients with prostate cancer.

## 5 CONCLUSIONS

Among patients with prostate cancer on active surveillance, we uncovered a range of perspectives experiences relating to the selection, interpretation and clinical impact of genomic testing. Although testing was useful to clinical decision‐making for some patients, there were meaningful gaps in communication about testing and knowledge of results. These findings can be used to inform interventions that improve the quality and outcomes of genomic testing for prostate cancer in clinical practice.

## AUTHOR CONTRIBUTIONS

**Michael S. Leapman** and **Dena Schulman‐Green:** Study conception; design; data collection; analysis; interpretation; manuscript preparation. **Ryan Sutherland:** Data collection; analysis; interpretation. **Cary P. Gross** and **Xiaomei Ma:** Study conception and design, manuscript preparation. **Tyler M. Seibert,** **Matthew R. Cooperberg,** **William J. Catalona** and **Stacy Loeb:** Data analysis and interpretation of results; manuscript preparation.

## ACKNOWLEDGEMENTS

None.

## CONFLICT OF INTEREST STATEMENT

ML, MRC and TMS report research collaborations with Veracyte Inc.
